# ConceptACT: episode-level concepts for sample-efficient robotic imitation learning

**DOI:** 10.3389/frobt.2026.1865290

**Published:** 2026-08-12

**Authors:** Jakob Karalus, Friedhelm Schwenker

**Affiliations:** 1 Institute of Artificial Intelligence, Ulm University, Ulm, Germany; 2 Institute of Neuroinformatics, Ulm University, Ulm, Germany

**Keywords:** action chunking, attention mechanisms, concept learning, human-robot interaction, imitation learning, robotic manipulation, sample efficiency, transformer networks

## Abstract

Imitation learning enables robots to acquire complex manipulation skills from human demonstrations, but current methods rely solely on low-level sensorimotor data while ignoring the rich semantic knowledge humans naturally possess about tasks. We present ConceptACT, an extension of Action Chunking with Transformers that leverages episode-level semantic concept annotations during training to improve learning efficiency. Unlike language-conditioned approaches that require semantic input at deployment, ConceptACT uses human-provided concepts (object properties, spatial relationships, task constraints) exclusively during demonstration collection, adding minimal annotation burden. We integrate concepts using a modified transformer architecture in which the final encoder layer implements concept-aware cross-attention, supervised to align with human annotations. Through experiments on two robotic manipulation tasks with logical constraints, we demonstrate that ConceptACT converges faster and achieves superior sample efficiency compared to standard ACT. Crucially, we show that architectural integration through attention mechanisms significantly outperforms naive auxiliary prediction losses or language-conditioned models. These results indicate that properly integrated semantic supervision provides useful inductive biases for manipulation tasks.

## 1 Introduction

In recent years, Imitation Learning has emerged as a successful technique for teaching robots complex skills by learning from expert demonstrations (Schaal, 1999; Argall et al., 2009). In robotic manipulation, these demonstrations are typically collected through teleoperation, where a human operator controls the robot to perform the task (Zhao et al., 2023; Mandlekar et al., 2022). The advantage of learning from demonstrations (in contrast to traditional reinforcement learning or other approaches) is that no explicit reward functions or control algorithms have to be designed. The approach of learning from (expert) trajectories makes it especially valuable for tasks where good behavior is easier to demonstrate than to formalize. However, current imitation learning methods share a fundamental limitation: they learn from raw sensory observations alone, whether low-dimensional robot-state signals such as joint positions and forces or high-dimensional camera images, without access to the explicit semantic knowledge that humans possess when demonstrating a task (e.g., Zare et al., 2024). Camera images carry rich spatial information, but the policy must still infer task-relevant concepts such as object properties and spatial relationships from them implicitly. The semantic structure is never provided directly.

This is in contrast to the way humans teach complex tasks to other humans. In these settings, humans naturally employ a variety of techniques to help the learner reach their goal. The umbrella term for these techniques in psychological research is “scaffolding,” a metaphor for the structural supports the learner receives during their skill-building phase. For example, techniques like providing conceptual frameworks, highlighting important features, and explaining underlying principles are natural for humans to use. But they are usually employed to accelerate the learning process (Van de Pol et al., 2010; Shao et al., 2023). In contrast, machine learning from demonstration (i.e., Imitation Learning) is typically a black box for the human, which learns implicit associations between high-dimensional observations and actions without access to the semantic reasoning that guides human decision-making (Firoozi et al., 2025). This mismatch represents a significant missed opportunity: human demonstrators possess valuable high-level knowledge about task structure, object properties, and causal relationships that could substantially improve learning efficiency if properly leveraged.

Current state-of-the-art approaches like ACT (Zhao et al., 2023) are limited to learning from low-level data, failing to gather the conceptual understanding that humans can readily provide alongside their demonstrations. This limitation can be especially pronounced in manipulation tasks that involve complex reasoning about object properties, spatial relationships, or task constraints, which are scenarios where semantic guidance could prove most beneficial.

**FIGURE 1:**
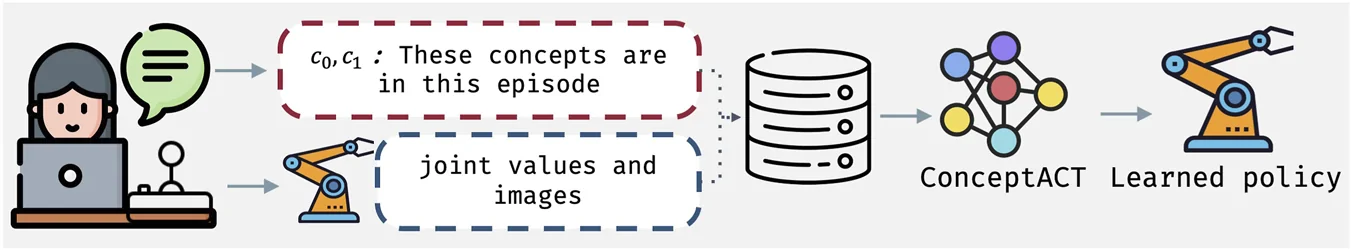
Overview of our approach: We enhance the normal Imitation Learning approach by allowing the user to specify which concepts are in an episode.

We address this limitation by introducing ConceptACT, an extension of the ACT architecture that integrates episode-level semantic concepts directly into the imitation learning process (Figure 1). Our approach leverages a key practical insight: human demonstrators naturally observe object properties, spatial relationships, and task constraints while performing demonstrations, making episode-level concept annotation straightforward and minimally expensive (in terms of annotation cost) during the collection of the demonstration data. In contrast to methods that require semantic descriptions or language instructions at deployment time, placing ongoing burdens on end users, ConceptACT requires concept information only during the one-time demonstration phase. Once training is complete, the resulting policy operates on standard low-level inputs without needing any additional semantic information during deployment.

Our technical approach enables human demonstrators to annotate episodes with high-level concepts (such as object colors, shapes, or spatial relationships) and uses these annotations as auxiliary supervision during training. By incorporating a modified *Concept Transformer* (Rigotti et al., 2022) architecture into the standard ACT encoder, ConceptACT learns to attend to semantically meaningful concepts while predicting action sequences, creating stronger inductive biases that improve sample efficiency and task understanding. We evaluate ConceptACT on two robotic pick-and-place tasks with high-level sorting or ordering constraints, where robots must manipulate objects of varying shapes and colors according to complex conditional rules.

The primary contributions of this work are threefold: (1) we demonstrate a systematic approach for integrating episode-level semantic concepts into transformer-based imitation learning, (2) we show that proper architectural integration of concepts through attention mechanisms provides superior learning compared to auxiliary prediction tasks, and (3) we provide empirical evidence that concept-guided learning improves sample efficiency in manipulation tasks requiring conditional reasoning about object properties.

## 2 Related work

Our work integrates high-level semantic concepts into imitation learning to improve sample efficiency and interpretability. Here, we review related approaches that incorporate auxiliary information, particularly concepts, into various learning paradigms.

Hristov and Ramamoorthy (2021) label demonstrated trajectories with high-level spatial concepts (“behind”, “on top”) and temporal concepts (“quickly”). Their method learns disentangled representations that separate task-relevant features from task-irrelevant variations. While they focus on trajectory-level semantic labeling similar to our episode-level concepts, their approach targets explainability through disentanglement rather than improving sample efficiency through auxiliary supervision.

Cubek et al. (2015) employs the conceptual spaces theory for learning from demonstration, using subspace clustering to identify relevant conceptual dimensions from sensory data. Their method automatically discovers conceptual representations that bridge low-level sensory input and high-level task understanding but relies on hard-coded high-level actions and leverages symbolic planning. Unlike their unsupervised concept discovery, ConceptACT uses human-provided concept annotations during training to guide the learning process explicitly.

Stepputtis et al. (2020) combine natural language, vision, and motion for abstract task representation in imitation learning. Their multi-modal approach learns policies conditioned on language instructions, enabling generalization across task variations. While both approaches integrate high-level semantic information, their language conditioning operates at inference time for task specification, whereas ConceptACT uses concept annotations during training to improve learning efficiency.

Ye et al. (2025) reduces concept annotation requirements in RL to 500–5000 labels through active learning and concept ensembles. They interleave concept learning with online RL training, where the agent explores and learns simultaneously. In contrast, ConceptACT operates in offline imitation learning with fixed demonstration datasets and episode-level annotations. We integrate concepts directly into the transformer architecture through attention mechanisms, whereas theirs maintains separate concept and policy networks.

Das et al. (2023) shows that generating concept-based explanations during training improves learning through the Protégé Effect. Their agents learn to produce natural language explanations grounded in predefined concepts as auxiliary outputs. ConceptACT instead uses human-provided concepts as auxiliary inputs, modifying the transformer’s internal attention to incorporate semantic information directly into action prediction rather than generating separate explanations.

Haramati et al. (2024) decomposes visual observations into entity-specific representations, enabling agents trained with 3 objects to generalize to 10+ objects. They automatically extract object-level structure through architectural inductive biases without human supervision. ConceptACT differs by incorporating human-defined semantic concepts at the episode level, explicitly leveraging domain knowledge about task structure rather than discovering it automatically.

The idea of restricting network architectures to operate through explicit concepts has been investigated extensively in supervised learning. These concept-based methods fall into two paradigms: *post hoc* explanation methods and inherently interpretable models. Post-hoc methods such as TCAV (Kim et al., 2018) analyze trained models to understand how user-defined concepts influence predictions without altering the model itself. Inherently interpretable models, by contrast, incorporate concepts directly into the architecture. Concept Bottleneck Models (Koh et al., 2020) route predictions through an interpretable concept layer, Concept Whitening (Chen et al., 2020) aligns latent representations with predefined concepts, ProtoPNet (Chen et al., 2019) learns prototypical concept representations, Self-Explaining Neural Networks (Alvarez Melis and Jaakkola, 2018) generate instance-level explanations as part of prediction, and Concept Transformer (Rigotti et al., 2022) replaces standard attention with concept-grounded cross-attention. These approaches were developed primarily for interpretability in classification tasks, though their architectures generalize beyond that setting. Our work adapts the Concept Transformer for a different purpose: improving learning efficiency in imitation learning through episode-level concept supervision.

Recent work demonstrates that concept-based auxiliary supervision can significantly improve learning performance. For instance, Fontana et al. (2024) show how auxiliary concept tasks enhance computer vision performance through multi-task learning, while Yao et al. (2023) demonstrate that semantic auxiliary tasks improve retrieval performance even with limited annotations. Similarly, Mahapatra et al. (2020) integrates semantic guidance into GAN learning, showing improved classification and segmentation performance. These works highlight that concept supervision provides not just interpretability but also serves as a powerful inductive bias that improves sample efficiency and generalization.

Human explanations have proven effective for accelerating learning across multiple reinforcement learning domains. Guan et al. (2021) demonstrated that factual explanations highlighting important image regions in Human-in-the-Loop RL significantly improve learning speed. Similarly, factual explanations of temporal information in preference-based RL accelerate the learning process (Holk et al., 2024; Karalus and Schwenker, 2025). Karalus and Lindner (2022) showed that integrating counterfactual explanations into the TAMER (Knox and Stone, 2009) framework reduces sample complexity. These findings consistently demonstrate that enabling humans to explain different aspects of a task improves both learning speed and sample efficiency.

Our ConceptACT approach is distinguished by its integration of semantic concept learning directly into the action chunking transformer architecture for imitation learning. Unlike methods that discover concepts unsupervised or use them solely for interpretability, we leverage human-provided concept annotations as auxiliary supervision to improve sample efficiency. Furthermore, while most concept-based approaches in RL focus on state abstraction or skill discovery, ConceptACT operates at the episode level, providing semantic guidance that helps the policy learn the underlying task structure more effectively. This positions our work at the intersection of interpretable machine learning and practical robotic imitation learning, addressing both the need for sample-efficient learning and human-understandable decision-making in robotic systems.

## 3 Background

We consider sequential decision-making problems in which an agent observes states $s_{t}$ and executes actions $a_{t}$ at each timestep, with the objective of learning a policy π:S→A from expert demonstrations rather than from a reward signal.

### 3.1 Behavior Cloning

Given a dataset of expert demonstrations $D={\left\{\left(s_{i},a_{i}\right)\right\}}_{i=1}^{N}$ consisting of state-action pairs collected from expert trajectories, the goal is to learn a policy $\pi_{\theta }$ parameterized by θ that mimics the expert’s behavior. The underlying assumption is that the expert demonstrations are generated by an optimal or near-optimal policy $\pi^{*}$ .

*Behavior Cloning* (BC) represents the most direct approach to imitation learning, in which the problem is formulated as supervised learning. The policy parameters θ are optimized to minimize the discrepancy between predicted and demonstrated actions:

$$L_{BC}\left(\theta \right)=E_{\left(s,a\right)∼D}\left[\ell \left(\pi_{\theta }\left(s\right),a\right)\right]$$

where ℓ(⋅,⋅) is typically the $L_{1}$ or $L_{2}$ norm for continuous action spaces.

### 3.2 Action chunking with transformers (ACT)

One of the more prominent recent advances in imitation learning, especially for robotic manipulation, is *ACT*: *Action Chunking with Transformers* (Zhao et al., 2023). ACT introduces three key architectural innovations: action chunking (predicting sequences rather than single actions), variational autoencoder training to encode style differences, and a transformer encoder-decoder architecture for multimodal input processing.

#### 3.2.1 Action chunking

Rather than predicting single actions at each timestep, ACT predicts action sequences:

$$\pi_{\theta }\left(a_{t:t+k-1}|s_{t}\right)=\pi_{\theta }\left(a_{t},a_{t+1},\ldots ,a_{t+k-1}|s_{t}\right)$$

#### 3.2.2 Variational training

ACT employs a variational autoencoder (Kingma and Welling, 2014) to capture trajectory style variation. A VAE-Encoder^1^ processes the sequence of joint variables as input and infers the posterior distribution over a latent style variable $z\in R^{L}$ :

$$\begin{array}{cc} q_{\phi }\left(z|s_{t},a_{t:t+k-1}\right) & =N\left(\mu_{\phi }\left(s_{t},a_{t:t+k-1}\right),\right.\\ & \left.\sigma_{\phi }^{2}\left(s_{t},a_{t:t+k-1}\right)\right) \end{array}$$

This latent variable z is then used as input for the VAE-Decoder. At inference time, z is set to zero, producing deterministic predictions.

#### 3.2.3 Transformer architecture

The VAE-Decoder integrates a transformer encoder-decoder to process multimodal inputs. The latent variable $z\in R^{L}$ sampled from the VAE-Encoder provides a compressed representation of the trajectory style, while current proprioceptive information is concatenated into a state vector $s_{t}\in R^{d_{s}}$ . Visual information from multiple camera viewpoints is processed through pre-trained ImageNet encoders (usually frozen ResNet (He et al., 2016) or ViT (Dosovitskiy et al., 2021) backbones). Each camera image $I_{i}\in R^{H\times W\times 3}$ produces feature maps $F_{i}\in R^{H^{\prime }\times W^{\prime }\times d}$ , which are tokenized by flattening the spatial dimensions into a sequence of visual tokens $\left\{f_{i,1},f_{i,2},\ldots ,f_{i,H^{\prime }W^{\prime }}\right\}$ where each token $f_{i,j}\in R^{d}$ represents a spatial patch.

The latent variable z and proprioceptive state $s_{t}$ are projected to the model dimension d through learned linear layers, creating tokens $z^{\prime }\in R^{d}$ and $s_{t}^{\prime }\in R^{d}$ . The complete encoder input sequence becomes:

$$X=\left[z^{\prime };s_{t}^{\prime };f_{i,1};f_{i,2};\ldots ,f_{i,H^{\prime }W^{\prime }}\right]\in R^{S\times d}$$

where $S=2+\sum_{i}H^{\prime }W^{\prime }$ represents the total sequence length across all modalities.

The transformer encoder processes this multimodal sequence through N identical layers, each containing multi-head self-attention followed by a position-wise feedforward network. For input sequence $X^{\left(l-1\right)}$ at layer l−1 , the self-attention is calculates as follows:

$${\text{Attention}}^{\left(l\right)}=\text{softmax}\left(\frac{Q^{\left(l\right)}K^{\left(l\right)T}}{\sqrt{d_{k}}}\right)V^{\left(l\right)}$$

With Q being: $Q^{\left(l\right)}=X^{\left(l-1\right)}W_{Q}^{\left(l\right)}$ , K and V are calculated similar with their respective weights $W_{K}$ and $W_{V}$ . The transformer decoder generates action sequences through cross-attention with the encoder output $X^{\left(N\right)}$ . The decoder uses learned positional embeddings for k action timesteps, initialized as $D_{in}\in R^{k\times d}$ . Each decoder layer calculates masked self-attention over action sequence positions, followed by cross-attention to the encoder output. The final action sequences (since ACT does action chunking, multiple actions need to be predicted) are generated through a learned linear layer, which takes the final decoder output $\left(D^{\left(L\right)}\in R^{k\times d}\right)$ as an input:

$$A_{t:t+k-1}=D^{\left(L\right)}W_{\mathrm{action}}\in R^{k\times d_{a}}$$

where $d_{a}$ represents the action dimensionality. During inference, ACT employs temporal ensembling to combine overlapping action chunks for smoother execution (Zhao et al., 2023).

#### 3.2.4 Complete training

The complete training objective combines action reconstruction with KL regularization of the latent variable:

$$\begin{array}{ll} L_{\mathrm{ACT}} & =E_{q_{\phi }}\left[\ell \left(a_{t:t+k-1},p_{\theta }\left(a_{t:t+k-1}|s_{t},z\right)\right)\right]\\ & \;+\beta \cdot D_{KL}\left[q_{\phi }\left(z|s_{t},a_{t:t+k-1}\right)\|N\left(0,I\right)\right] \end{array}$$

where ℓ(⋅,⋅) is typically the L1 loss for continuous actions and β controls the trade-off between reconstruction accuracy and latent regularization.

#### 3.2.5 LAV-ACT: language-augmented visual action chunking

Tripathi et al. (2025) extend ACT by incorporating textual task descriptions alongside visual and proprioceptive inputs, similar to Visual Language Action Models (VLAs) (Kim et al., 2025). LAV-ACT modifies the standard ACT backbone through a dual-stream architecture that processes visual and linguistic information separately before fusion.

The architecture replaces the standard ResNet (He et al., 2016) visual encoder with a multimodal processing pipeline. Visual features are extracted using ResNet, while language descriptions are encoded through a Voltron encoder (Karamcheti et al., 2023). These separate embeddings are then fused using Feature-wise Linear Modulation (FiLM) layers (Perez et al., 2018), which allow language features to modulate visual representations through learned affine transformations. The resulting multimodal embeddings serve as inputs to the standard ACT VAE-Decoder, enabling the policy to condition action predictions on both visual observations and linguistic task specifications.

This language conditioning enables task generalization across different verbal instructions while maintaining the temporal consistency benefits of action chunking. However, LAV-ACT still operates solely on low-level sensorimotor data augmented with natural language, without incorporating the structured semantic concepts that ConceptACT leverages for improved sample efficiency.

### 3.3 Concept transformers

*Concept Transformer* by Rigotti et al. (2022) modifies the attention mechanism so that attention operates over human-defined concepts rather than input tokens. In standard attention (Vaswani et al., 2017), queries, keys, and values are all derived from the input sequence through learned linear transformations. The Concept Transformer replaces the input in the computation of keys and values with learnable concept embeddings, while queries remain unchanged.

Given input features $X\in R^{P\times d}$ (with P patches or tokens and d embedding dimension) and a set of C concept embeddings $c_{1},\ldots ,c_{C}\in R^{d}$ organized as $C=\left[c_{1};\ldots ;c_{C}\right]\in R^{C\times d}$ , the attention computation becomes:

$$Q=XW_{Q}$$

$$K=CW_{K}$$

$$V=CW_{V}$$

The attention weights are computed as:

$$\alpha_{sc}=\text{softmax}{\left(\frac{QK^{T}}{\sqrt{d}}\right)}_{sc}$$

where s indexes input positions and c indexes concepts. This produces an attention matrix $A=\left[\alpha_{sc}\right]\in R^{S\times C}$ where each entry represents how much input position s attends to concept c .

During training, human experts provide ground-truth concept annotations $H\in R^{P\times C}$ , where $H_{pc}=1$ indicates that concept c is present at input position p . The concept supervision loss aligns the learned attention with these annotations using the squared Frobenius norm:

$$L_{\mathrm{concept}}=\|A-H{\|}_{F}^{2}=\overset{P}{\underset{p=1}{\sum }}\overset{C}{\underset{c=1}{\sum }}{\left(\alpha_{pc}-H_{pc}\right)}^{2}$$

## 4 Implementation

In this section we present ConceptACT, which is the core contribution of this work. While we build on the previously presented ACT architecture and extend it to include the Concept Transformer our unique contribution lies in three parts: First, the Concept Transformer is extended to a class-aware concept attention that maintains separate embeddings and supervision for each concept class. Second, its integration for the final ACT encoder layer, with a residual stream. Third, the definition and usage of episode-level concept supervision that is required only during training and adds no inference cost.

We extend the standard imitation learning setting to incorporate high-level semantic information that humans naturally use when teaching tasks. The high-level goal is to allow humans to formulate knowledge about the current episode and include this information in the training process.

Crucially, episode-level concept annotations are required only during the demonstration phase. Human demonstrators naturally observe object properties and task constraints while performing demonstrations, making episode-level annotation straightforward and non-intrusive. Once training is complete, the resulting policy operates on normal inputs without requiring any concepts at deployment time. A further practical advantage of episode-level concepts are that these are often easy targets for automatic extractions. For example, the structured demonstrations plan: 10 red cube episodes, then 10 blue cylinder episodes already contains concepts about shape and color. This positions ConceptACT not only as compatible with manual annotation workflows but also as a candidate for fully automated concept integration in settings where environmental metadata is programmatically accessible.

### 4.1 Problem formulation with concepts

In addition to the demonstration dataset $D={\left\{\left(s_{i},a_{i}\right)\right\}}_{i=1}^{N}$ of state-action pairs, we assume access to concept annotations produced by humans during the demonstration phase. Rather than creating a single flat concept vector, we maintain separate concept annotations for each concept class, better reflecting the semantic structure of the problem.

We define a set of concept classes $T=\left\{T_{1},T_{2},\ldots ,T_{K}\right\}$ which represent higher-level concept groups, such as object colors, shapes, or spatial relationships. Each concept class $T_{j}$ contains a finite set of mutually exclusive concept values representing the concrete instantiation of that concept in the current episode. For example, a color concept class contains $T_{\text{color}}=\left\{\text{red},\text{green},\text{blue},\text{yellow}\right\}$ as potential concept values.

For each episode e and concept class $T_{j}$ , we define:

$$c^{\left(e\right),T_{j}}\in {\left\{0,1\right\}}^{\left|T_{j}\right|}$$

where $c^{\left(e\right),T_{j}}$ is a one-hot encoding indicating which specific concept value within concept class $T_{j}$ is present in episode e . The complete concept annotation for episode e is thus represented as the set:

$$C^{\left(e\right)}=\left\{c^{\left(e\right),T_{1}},c^{\left(e\right),T_{2}},\ldots ,c^{\left(e\right),T_{K}}\right\}$$

**Example:** A episode for a manipulation tasks contains a blue objects, with a rough surface (important for gripping) and it should be dropped in “zone B”, with the following concept classes: $T_{\text{color}}=\left\{\text{red},\text{green},\text{blue},\text{yellow}\right\}$ , $T_{\text{shape}}=\left\{\text{cube},\text{cylinder},\text{sphere}\right\}$ , $T_{\text{surface}}=\left\{\text{smooth},\text{rough}\right\}$ , and $T_{\text{zone}}=\left\{\text{A},\text{B},\text{C}\right\}$ , the annotations would be:

$$\begin{array}{ll} c^{\left(e\right),T_{\text{color}}} & =\left[0,0,1,0\right]\\ c^{\left(e\right),T_{\text{shape}}} & =\left[1,0,0\right]\\ c^{\left(e\right),T_{\text{surface}}} & =\left[0,1\right]\\ c^{\left(e\right),T_{\text{zone}}} & =\left[0,1,0\right] \end{array}$$

Since policy training operates at the step level, we assign each timestep the concept annotation of its parent episode: all steps within episode e share the same concept vector $c_{t}^{T_{j}}=c^{\left(e\right),T_{j}}$ . This constant mapping holds by design, as humans annotate concepts once per episode.

### 4.2 ConceptACT architecture

**FIGURE 2:**
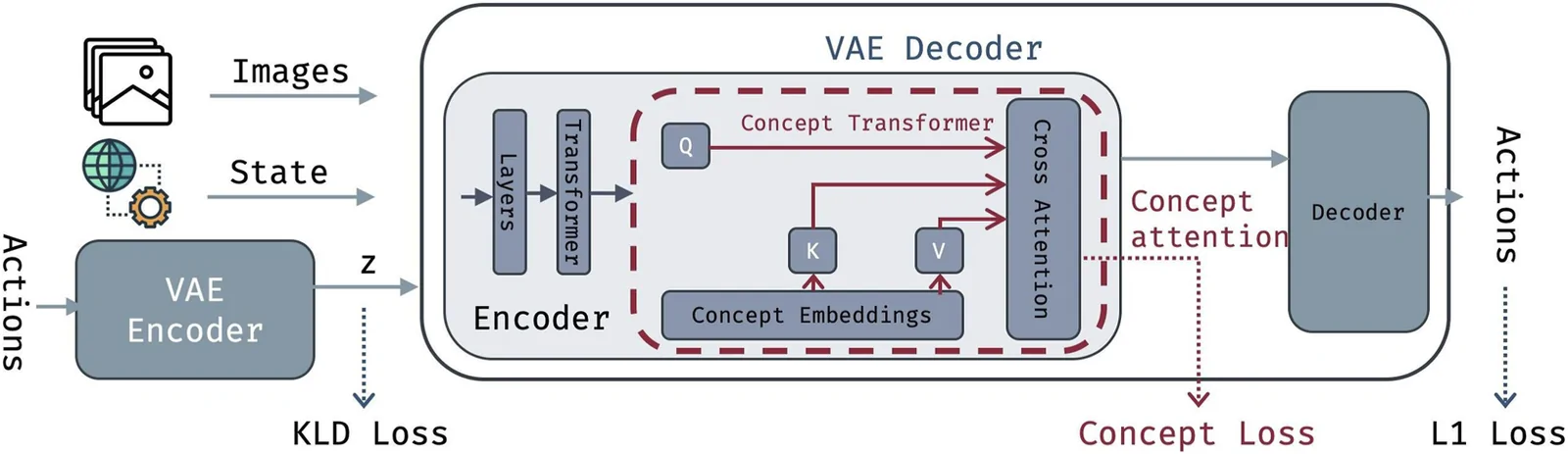
Technical overview of our approach: We integrate these concepts by extending the ACT architecture to include a concept transformer that aligns its attention mechanism with the given concepts. This alignment cost can then be included in the total loss. Red indicates changes.

To incorporate concept learning into ACT training, we modify the architecture by replacing the final layer of the transformer encoder within the VAE-Decoder component. All other components of the ACT architecture, including the VAE-Encoder, transformer decoder, and action prediction head, remain unchanged, ensuring that our modifications are minimal and focused. A technical overview of our architecture can be viewed in Figure 2.

Our decision to modify the transformer encoder (within the VAE decoder) is motivated by several architectural considerations. The VAE-Encoder processes only proprioceptive joint information and action sequences to maintain computational efficiency, excluding visual inputs that are essential for learning image-based concepts, such as object colors and shapes. The transformer decoder, while powerful for sequential action prediction, typically consists of only a single layer and is specifically designed for the generative task of producing action sequences, making it less suitable for the discriminative concept classification task.

In contrast, the transformer encoder within the VAE-Decoder receives the complete multimodal input, including visual features from multiple camera viewpoints, proprioceptive state information, and latent variables, providing the rich representational foundation necessary for concept learning. By positioning concept learning at the final encoder layer, we ensure that concept information is learned from the highest-level multimodal representations while still being able to influence the features passed to the action decoder.

A core feature of our contributions is that our concept annotations only change the learning phase of the ACT framework. During inference (i.e., deployment), no knowledge of concepts is needed to use the policy to predict actions. This is a core strength of the presented approach. While the annotation of concepts during data-collection is a one-time task and therefore cheap, an annotation of concepts at the inference (i.e., deployment) stage would require continued online labelling effort by the human, which would be costly and impractical in most Imitation Learning deployments.

#### 4.2.1 Class-aware concept transformer integration

To integrate concept into the ACT rchitecture we replace the final layer of transformer encoder with a (customized) concept transformer. We deviate from the normal concept transformer forumulation because in our setup we have more information: the class structure of concepts. Rather than treating all concepts uniformly as in standard concept transformer approaches, our class-aware architecture has separate learnable concept embeddings for each concept class $T_{j}$ . For a concept class $T_{j}$ with cardinality $\left|T_{j}\right|$ (representing the number of possible values within that class), we define concept embeddings:

$$E_{j}\in R^{|T_{j}|\times d}$$

where d is the embedding dimension and each row represents a learnable embedding for one concept value within class $T_{j}$ .

Given encoder input $X\in R^{S\times d}$ where S is the sequence length (including tokens from images, proprioceptive state, and other modalities), each concept class $T_{j}$ computes its own attention mechanism through class-specific projection matrices:

$$Q_{j}=XW_{Q}^{\left(j\right)}$$

$$K_{j}=E_{j}W_{K}^{\left(j\right)}$$

$$V_{j}=E_{j}W_{V}^{\left(j\right)}$$

where $W_{Q}^{\left(j\right)},W_{K}^{\left(j\right)},W_{V}^{\left(j\right)}\in R^{d\times d}$ are learned projection matrices specific to concept class $T_{j}$ , and $E_{j}\in R^{|T_{j}|\times d}$ are the learnable concept embeddings for class $T_{j}$ .

The attention weights for concept class $T_{j}$ are computed as:

$$\alpha_{sc}^{\left(j\right)}=\text{softmax}{\left(\frac{Q_{j}K_{j}^{T}}{\sqrt{d}}\right)}_{sc}$$

where the subscript s indexes sequence positions (s=1,…,S) and c indexes the concepts within class $T_{j}$ $\left(c=1,\ldots ,|T_{j}|\right)$ . Due to the specific dimensionality of concept embeddings $E_{j}$ , the attention weight matrix $\alpha^{\left(j\right)}$ has shape $S\times |T_{j}|$ rather than the typical sequence length by embedding dimension. This change is crucial since in later steps the attention weights are used for a supervised concept loss.

#### 4.2.2 Concept transformer output computation

For each sequence position s and concept class $T_{j}$ , the concept transformer produces an output by computing the attention-weighted combination of concept value vectors:

$${\text{o}}_{s}^{\left(j\right)}=\overset{\left|T_{j}\right|}{\underset{c=1}{\sum }}\alpha_{sc}^{\left(j\right)}V_{jc}$$

where $V_{jc}\in R^{d}$ represents the c -th value vector (corresponding to the c -th concept within class $T_{j}$ ). This operation yields ${\text{o}}_{s}^{\left(j\right)}\in R^{d}$ for each sequence position s .

To integrate concept information with the original sequence representation, we concatenate the concept outputs from all classes with the original input for each sequence position:

$$X_{s}^{\text{enhanced}}=\left[X_{s};{\text{o}}_{s}^{\left(1\right)};{\text{o}}_{s}^{\left(2\right)};\ldots ;{\text{o}}_{s}^{\left(K\right)}\right]$$

where $X_{s}^{\text{enhanced}}\in R^{d\cdot \left(K+1\right)}$ represents the enhanced representation for sequence position s that combines original features with concept-aware representations from all K concept classes. This is a slight modification from the original concept transformer, where the is purely computed from a weighted sum of concepts. Here, we introduce a residual connection $X_{s}$ , which allows the decoder upstream to theoretically ignore the output of the concept transformer.

A projection layer then maps this concatenated representation back to the original embedding dimension:

$$Y_{s}=W_{\text{proj}}X_{s}^{\text{enhanced}}+b_{\text{proj}}$$

where $W_{\text{proj}}\in R^{d\times d\left(K+1\right)}$ and $b_{\text{proj}}\in R^{d}$ are learned parameters. The resulting sequence $Y=\left[Y_{1};Y_{2};\ldots ;Y_{S}\right]\in R^{S\times d}$ maintains the original dimensionality and serves as input to the ACT decoder.

#### 4.2.3 Concept prediction & loss formulation

While the sequence Y serves as the major output of the concept transformer, the more crucial change of our architecture is the usage of the auxiliary concept attention $\alpha_{sc}^{\left(j\right)}$ , which is used further to align the concept transformer with the human concepts. The encoder input sequence X contains tokens representing various modalities that collectively describe a single timestep in the demonstration trajectory. Although we process this information as a sequence of tokens for computational efficiency, it conceptually represents a unified observation at one demonstration step. Therefore, we must aggregate across the sequence dimension to produce a single concept prediction per class for each demonstration step.^2^

We obtain concept predictions for class $T_{j}$ through mean pooling across sequence positions:

$${\hat{c}}^{T_{j}}=\frac{1}{S}\overset{S}{\underset{s=1}{\sum }}\alpha_{sc}^{\left(j\right)}$$

This mean pooling operation transforms the sequence-level concept representations into a single prediction vector for each concept class, ensuring that our final concept predictions correspond to the episode-level annotations provided by human demonstrators.

Our concept loss combines cross-entropy losses across all concept classes^3^:

$$L_{\text{concept}}^{\text{CE}}=\overset{K}{\underset{j=1}{\sum }}\text{CrossEntropy}\left({\hat{c}}^{T_{j}},\;c^{T_{j}}\right)$$

where ${\hat{c}}^{T_{j}}$ represents the predicted logits for concept class $T_{j}$ , obtained from mean pooling as described above, and $c^{T_{j}}$ is the ground-truth one-hot annotation.

**Worked Example.** To ground the above formulation, we trace tensor dimensions through the color concept class ( $|T_{\text{color}}|=4$ values: red, green, blue, yellow) for an episode containing a *red cube*. The human annotation is $c^{T_{\text{color}}}=\left[1,0,0,0\right]$ . Idealized PyTorch pseudocode for the calculation of concept attention of the concept colors:

The key dimensional asymmetry occurs at the scores computation: queries have S rows (input tokens) while keys have $\left|T_{j}\right|$ rows (concept values), producing an $S\times |T_{j}|$ attention matrix. Mean pooling across S then yields a prediction vector in $R^{\left|T_{j}\right|}$ that matches the human one-hot annotation exactly, enabling direct cross-entropy supervision.

#### 4.2.4 Complete training objective

The final ConceptACT training objective integrates the class-aware concept loss with the standard ACT components:

$$L_{\text{total}}=L_{\mathrm{ACT}}+\lambda_{\text{concept}}L_{\text{concept}}^{\text{CE}}$$

where $L_{\mathrm{ACT}}$ encompasses both action reconstruction loss and KL divergence regularization as defined in the standard ACT formulation.

This class-aware concept transformer layer replaces the final encoder layer within the VAE-Decoder component, positioning concept learning at the highest representational level before action prediction. The architecture addresses three specific limitations of standard concept transformers in our setting. First, the separate attention mechanisms per concept class eliminate competition between semantically unrelated concepts (e.g., colors competing with shapes for attention), allowing each class to develop specialized representations. Second, the variable-dimension concept embeddings $E_{j}\in R^{|T_{j}|\times d}$ accommodate concept classes with different cardinalities without requiring padding or masking operations that could interfere with learning. Third, the cross-entropy loss per class enforces the constraint that exactly one concept per class should be active, matching the one-hot structure of our human annotations and preventing the model from learning spurious multi-label predictions within a single concept class.

After training is complete, the concept prediction components can be discarded during inference, allowing the policy to be used normally for action prediction. This ensures that concept learning aids training without affecting deployment efficiency.

## 5 Evaluation

While ConceptACT could in principle apply to other imitation learning domains, we evaluate our approach in a robotic manipulation setting where concept-based guidance is well suited. Robotic tasks naturally exhibit complex state-action relationships that benefit from semantic reasoning, while their visual and proprioceptive observations enable intuitive concept definitions around object properties and spatial relationships.

We structure our evaluation around two complementary research questions that address the core bottlenecks in practical imitation learning: (1) *Sample efficiency*: Can concept supervision reduce the number of human demonstrations required to achieve target performance? (2) *Learning efficiency*: Can concept supervision accelerate convergence with fixed demonstration data? These questions correspond to the two primary cost factors in deploying imitation learning systems: human time for data collection and computational resources for training.

To address these questions systematically, we design two manipulation tasks that require different types of concept reasoning while maintaining consistent annotation methodology. Our experimental approach isolates the effect of concept integration by comparing ConceptACT variants against standard ACT across multiple data regimes and training horizons.

### 5.1 Hardware configuration

Our experimental setup utilizes a bilateral robotic system comprising two identical SO-100 robotic arms (Knight et al., 2024). One arm serves as the leader for human demonstration and teleoperation, while the other functions as the follower for policy execution and evaluation. This leader-follower configuration enables intuitive data collection: human demonstrators directly manipulate the leader’s arm through kinesthetic teaching, with joint positions and trajectories recorded in real-time.

Concept classes extracted automatically from the demonstration protocol at zero additional annotation cost.

The system is equipped with two cameras, providing complementary viewpoints: a gripper camera attached to the end-effector of the follower arm for detailed manipulation views, and a scene camera positioned to capture the entire workspace from a fixed overhead perspective. This dual-camera setup ensures comprehensive visual coverage of both fine-grained manipulation details and global scene context. Figure 4 shows the experimental setting. The Supplementary Material contains videos from both scene and gripper camera of multiple episodes.

### 5.2 Task description and concept annotations

We evaluate ConceptACT on two robotic manipulation tasks that require logical reasoning beyond simple pick-and-place motions. Both tasks share similar motion primitives but differ in their underlying constraint structures, validating the generality of our approach across manipulation scenarios.

**FIGURE 3:**
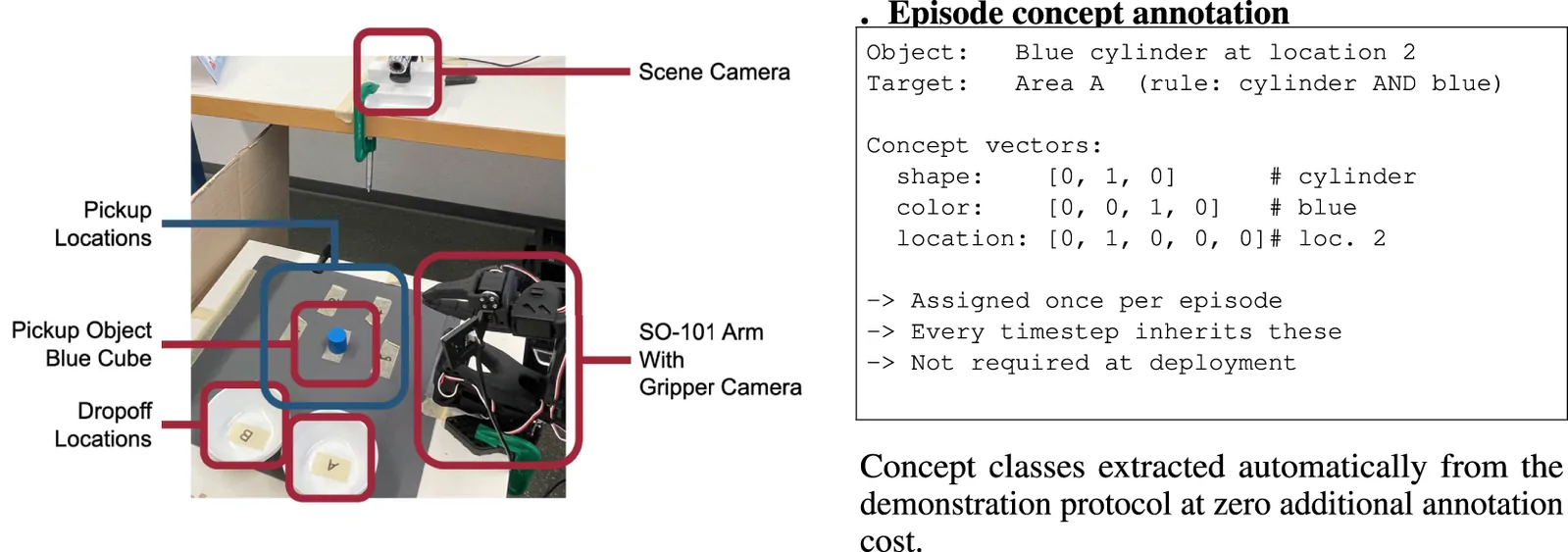
Task 1 (Sorting) setup and concept annotation. *Left:* Scene camera view showing the workspace with a blue cylinder, two collection areas, and the SO-100 follower arm. *Right:* The corresponding episode-level concept annotation as provided to ConceptACT during training. Each concept class is encoded as a one-hot vector. The sorting rule directs blue cylinders to Area A.

Task 1 (Sorting): The robot must grasp objects of varying shapes (cube, cylinder, rectangular prism) and colors (red, green, blue, yellow) from randomized positions and sort them into two collection areas according to conditional rules combining both attributes. The sorting rule creates a logical disjunction where objects are directed to Area A if they satisfy specific shape-color combinations, with all other objects sorted to Area B (see Supplementary Appendix 5.1 for complete task specification and sorting rules). Figure 3 shows the task setup and an example concept annotation for it.

**FIGURE 4:**
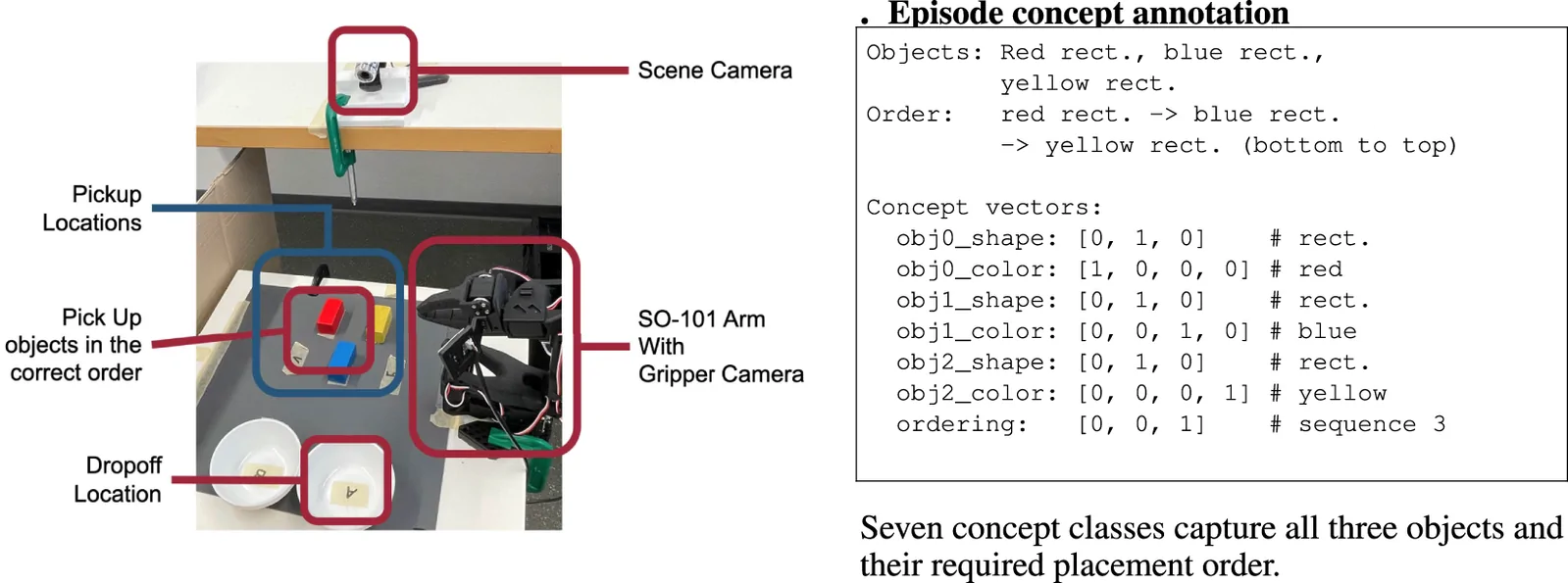
Task 2 (Ordering) setup and concept annotation. *Left:* Scene camera view showing three rectangles (red, blue, yellow) at separate pickup zones and the single collection area. *Right:* The episode-level concept annotation encoding shape, color, and ordering for all three objects. The color hierarchy ({red}≺yellow≺blue) determines the valid bottom-to-top sequence.

Task 2 (Ordering): Sequential placement of three objects into a single collection area according to multi-constraint optimization rules. The robot must satisfy simultaneous constraints on object cardinality, positional placement, color hierarchy, and adjacency relationships (see Supplementary Appendix 5.2 for the complete constraint system and longer task description). Figure 4 shows the task setup and an example concept annotation for it.

Currently, most work in Imitation Learning is usually only evaluated on a binary success/failure metric (Zhao et al., 2023; Chi et al., 2025; Brohan et al., 2022). This is due to the general approach of Imitation Learning, which focuses more on understanding higher-level tasks rather than exact task repetition. But a binary metric can mask learning (or problems in learning) in more complex tasks. Due to this, our evaluation improves on this standard through hierarchical scoring that decomposes task completion into distinct stages. For Task 1, we use a four-level metric (∈[0,3]) that distinguishes gripping failure (0), placement failure (1), correct placement but incorrect sorting (2), and complete success (3). For Task 2, a similar seven-level metric (∈[0,6]) is used. (See Supplementary Appendix 5 for more details). This approach follows recent benchmarks such as *ROBOEVAL* (Wang et al., 2025) and *FurnitureBench* (Heo et al., 2025), which promote similar metrics over binary outcomes. The important aspect of our hierarchical scoring systems is that they separate motor failures (scores 0–1) from logic failures (scores 2), directly showing whether a policy has learned the underlying task logic. For Task 1, we evaluate on 10 held-out test cases across 10 independently seeded models, yielding 100 physical robot trials per condition. Similar to Task 2 are 6 unseen test cases, resulting in 60 real human evaluations per condition.

Human demonstrators collected episodes at 50 Hz, recording joint positions, camera feeds, and timestamped actions. Each episode was annotated with concept vectors as one-hot encodings: for Task 1, shape $\left(c^{\text{shape}}\in {\left\{0,1\right\}}^{3}\right)$ , color $\left(c^{\text{color}}\in {\left\{0,1\right\}}^{4}\right)$ , and pickup location $\left(c^{\text{location}}\in {\left\{0,1\right\}}^{5}\right)$ ; for Task 2, shape and color for each of three objects plus ordering sequence. The required concept annotations for our changes were extracted automatically from the demonstration protocol. Since imitation learning data collection is typically structured around a plan, which ensures that the demonstrations are diverse enough for the tasks, which contains a lot of configuration effort. In our settings, the demonstration plan already contains all the needed concept information. Therefore, zero additional hand labeling was needed for the experiments.

For each plot and metric (Probability of Improvement and Optimality Gap), we calculate the mean and confidence intervals. For the metrics, these are computed via stratified bootstrapping (see Agarwal et al., 2021).

Seven concept classes capture all three objects and their required placement order.

#### 5.2.1 Baseline methods and implementation details

We compare five methods to isolate different aspects of concept integration:

- ACT: Standard baseline without concept information
- ConceptACT-Transformer: Full concept transformer integration (our primary method)
- ConceptACT-Heads: Architectural ablation using simple prediction heads (see Supplementary Appendix 1 for a detailed description). The goal of this ablation is to show that not only is the information about concepts important, but also our integration through the Concept Transformer is crucial.
- LAV-ACT - Specific: The LAV-ACT architecture receives task descriptions during both training and inference. In the “Specific” configuration, the model receives detailed task descriptions that contain information about the current scenario’s object properties and constraints, which our ConceptACT methods provide only during training.
- LAV-ACT - Generic: Since detailed task descriptions at inference time represent privileged information unavailable in practical deployments, we test LAV-ACT with generic task descriptions that leave out specific object properties. For example, we replace “Pick up the red cube and place it into zone A” with “Pick up the object and place it in the desired zone.” This configuration provides a more realistic comparison, as detailed semantic descriptions are costly to obtain at deployment time compared to the one-time annotation cost during demonstration collection.

We also evaluated LAV-ACT with an empty task string to simulate deployment without language input. This configuration failed to produce good policies because the FiLM conditioning layers require meaningful language embeddings to compute affine transformations over visual features. Without valid language input, the policy performs badly. The generic description variant, therefore, represents the closest approximation to a fair comparison.

A key practical distinction is that ConceptACT methods use concepts only during training, while LAV-ACT requires language descriptions at deployment time. This difference has significant implications for real-world applicability. ConceptACT, therefore, incurs no additional annotation cost after the demonstration phase, whereas LAV-ACT requires language descriptions for every deployment episode.

All models use identical hyperparameters, which were collected from the reference literature or implementation. For a full list of hyperparameters, please see Supplementary Appendix 3.

### 5.3 Sample efficiency experiments

We evaluate whether concept supervision reduces the number of human demonstrations required to achieve target performance. Using both manipulation tasks, we train models on varying dataset sizes (33%, 50%, 66%, 83%, and 100% of available demonstrations) and measure real robot performance on 10 held-out test episodes. Each configuration uses 10 random seeds to ensure statistical reliability.

#### 5.3.1 Task 1: sorting with limited demonstrations

**FIGURE 5:**
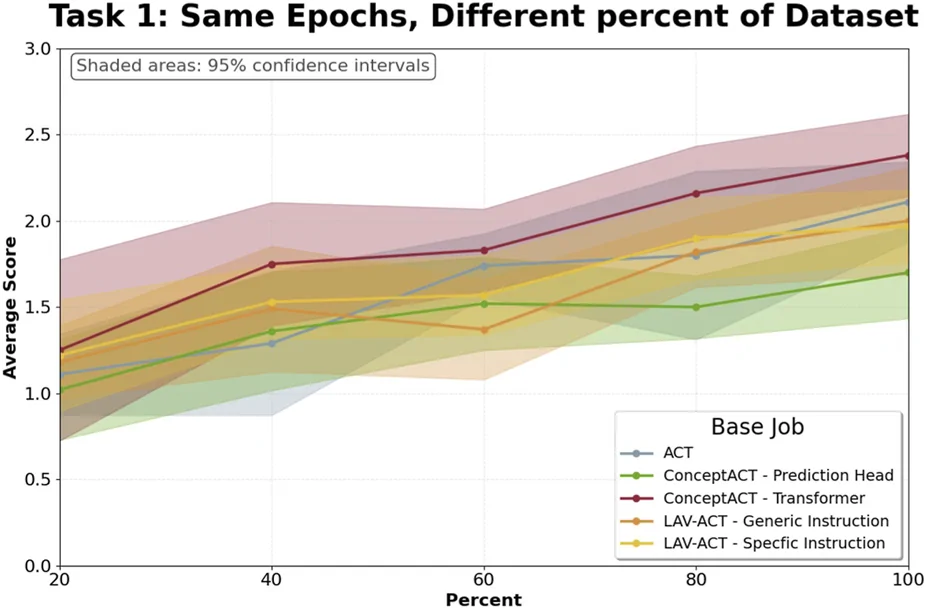
Sample efficiency results on task 1. Real robot evaluation performance is measured on different sizes of the dataset (with respect to the maximum size) on 10 test episodes (higher is better). Each method was trained with 10 random seeds; bold lines represent means, shaded areas show variance.

Figure 5 demonstrates that ConceptACT-Transformer achieves superior performance across all dataset sizes, with particularly pronounced advantages in low-data regimes. The method reaches near-optimal performance with only 50% of demonstrations, while standard ACT requires the full dataset to achieve comparable results.

#### 5.3.2 Task 2: ordering with limited demonstrations

**FIGURE 6:**
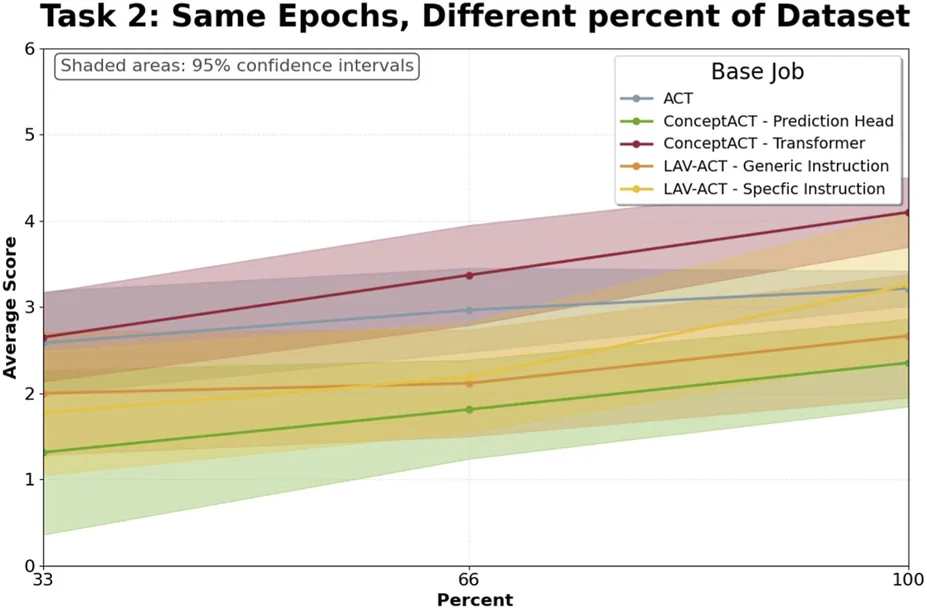
Sample efficiency results on task 2. Real robot evaluation performance is measured on different sizes of the dataset (with respect to the maximum size) on 6 test episodes (higher is better). Each method was trained with 10 random seeds; bold lines represent the means, and shaded areas show the variance.

The ordering task amplifies the benefits of concept guidance due to its increased conceptual complexity, which requires simultaneous reasoning about multiple objects and sequential constraints. As shown in Figure 6, ConceptACT-Transformer maintains consistent performance advantages across all data regimes, while the prediction head ablation (ConceptACT-Heads) performs poorly, particularly degrading as concept complexity increases.

The overlapping confidence intervals in both plots necessitate statistical analysis to quantify performance differences. Table 1 summarizes results using Probability of Improvement calculated via stratified bootstrapping. ConceptACT-Transformer achieves statistically significant performance gains across both tasks, with robust improvements over the architectural ablation. Notably, our approach outperforms both LAV-ACT variants despite using fewer parameters (see 6), demonstrating that architectural integration of concepts provides superior sample efficiency compared to language conditioning approaches. Importantly, ConceptACT achieves these performance improvements without requiring any additional information beyond standard sensorimotor inputs at the time of deployment.

**TABLE 1:** Sample efficiency results: probability of improvement aggregated across full training trajectory (higher is better).

Method	Probability of improvement					[95% CI]
ConceptACT - transformer	Task 1		Task 2		Both	
> ACT	0.64	[0.53, 0.75]	0.70	[0.57, 0.83]	0.66	[0.58, 0.75]
> LAV-ACT - specifc	0.65	[0.54, 0.76]	0.81	[0.67, 0.91]	0.71	[0.63, 0.79]
> LAV-ACT - generic	0.71	[0.60, 0.81]	0.83	[0.72, 0.92]	0.75	[0.72, 0.83]
> ConceptACT - heads	0.79	[0.69, 0.87]	0.93	[0.83, 1.00]	0.84	[0.77, 0.90]
ConceptACT - heads	Task 1		Task 2		Both	
> ACT	0.31	[0.21, 0.43]	0.13	[0.04, 0.25]	0.24	[0.17, 0.32]

Confidence intervals computed via bootstrapping.

The poor performance of ConceptACT-Heads, especially on the concept-rich Task 2, validates our architectural design choice. Simply adding concept prediction losses without modifying attention mechanisms fails to provide meaningful benefits and can even hinder performance when concept complexity increases.

### 5.4 Learning efficiency experiment

To address our second research question, whether concept supervision accelerates convergence with fixed demonstration data, we examine training dynamics across the full 10,000-step training horizon using 100% of available demonstrations. This analysis focuses on learning efficiency: can concept learning reduce the training time required to achieve target performance levels?

Using the sorting task (Task 1), we track both test-loss convergence and robot evaluation performance throughout training, measuring how quickly each method approaches its final performance. This experiment isolates the learning benefits of concept supervision from sample efficiency gains, addressing scenarios where demonstration data is abundant but training time remains a constraint.

**FIGURE 7:**
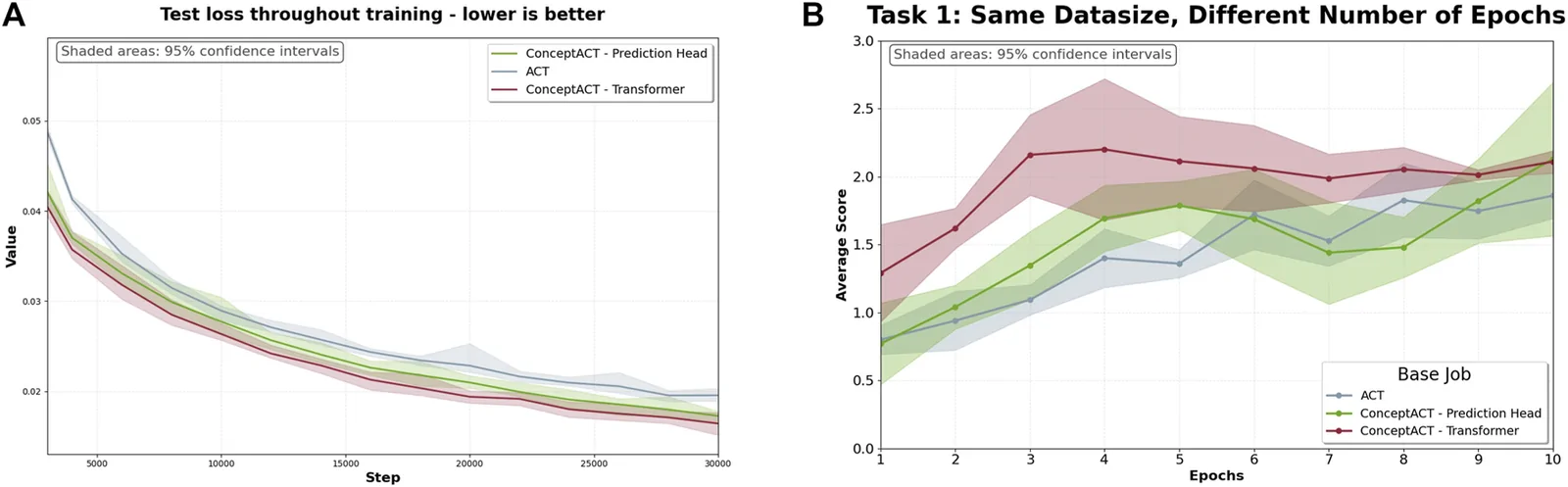
Learning efficiency results comparing convergence speed across methods. **(A)** Test loss on 20% holdout set throughout training (lower is better). **(B)** Real robot evaluation performance is measured every 3,000 training steps on 10 test episodes (higher is better). Each method was trained with 5 random seeds; bold lines represent means, shaded areas show variance.

As shown in Figure 7, ConceptACT (with Concept Transformer integration) demonstrates faster convergence during early training phases. This acceleration is evident in both test loss metrics (Figure 7A) and real robot evaluation performance (Figure 7B). The Concept Transformer method achieves its final performance approximately 6,000 steps earlier than standard ACT, resulting in a 40% reduction in training time to achieve comparable results.

The performance gap between methods becomes less significant in later training phases, which we attribute to the relative simplicity of the current task given the available demonstration data. Nevertheless, ConceptACT’s faster convergence provides benefits in scenarios where training time is high or rapid deployment is required.

The prediction head variant again yields minimal improvement over standard ACT, reinforcing our finding that the architectural integration method critically determines the effectiveness of concept supervision. This consistent pattern across both sample efficiency and learning efficiency experiments demonstrates that naive concept inclusion provides negligible benefits regardless of data regime.

Qualitatively, we observed distinct failure modes that reveal the underlying learning differences. Standard ACT policies frequently exhibited boundary confusion, dropping objects precisely at the border between collection areas or balancing them on edges, indicating successful manipulation learning but failure to internalize the sorting logic. ConceptACT policies never displayed this behavior, suggesting more robust acquisition of the underlying task structure encoded in concept annotations.

To quantify these learning efficiency gains, we computed optimality gaps across the full training trajectory and report aggregate performance with 95% confidence intervals using bootstrapping. Table 2 demonstrates that while both concept-based methods reduce the optimality gap, only the Concept Transformer integration achieves statistically significant improvement over baseline ACT.

**TABLE 2:** Learning efficiency results: Optimality gap aggregated across full training trajectory (lower is better).

Method	Optimality gap	95% CI
ACT	0.549	[0.48, 0.58]
ConceptACT - heads	0.515	[0.45, 0.56]
ConceptACT - transformer	0.317	[0.27, 0.40]

Confidence intervals computed via bootstrapping across 5 random seeds.

The results of these experiments show that our approach to incorporating a supervised loss (about concepts) improves learning efficiency. The 42% reduction in the optimality gap for our method (ConceptACT) can be important in practical deployment scenarios where computational resources are limited (e.g., on-device robotics).

### 5.5 Further experiments and ablations

These experiments are performed in simulation, which allows us to evaluate agents automatically instead of manually measuring performance in the real world, as done in the previous experiments. For brevity, we summarize each experiment with the Probability of Improvement metric (over the Optimality Gap), as in Table 2.

#### 5.5.1 Robustness to concept noise

Since the concept annotations in our setting are created by humans, there is a chance that they are noisy or incorrect. We argue that collecting annotations during the demonstration phase, and therefore with less time pressure, reduces this risk, but errors remain possible. To simulate this, we trained multiple models with different levels of noise added to the concept annotations. The noise respects the given class structure and its one-hot encoding.

The results (Table 3) show that ConceptACT is relatively stable under minor noise in the annotations. For larger noise, a small reduction is visible, but ConceptACT still performs solidly. These results show for uncorrelated noise, ConceptACT stays stable. While these results show only stability for random errors, not more biased mistake that human could make, they still provide some confidence for real world deployment.

**TABLE 3:** Probability of Improvement under noisy concept annotations against ACT. (higher is better).

Method	Probability of improvement	95% CI
0.0	0.74	[0.64, 0.83]
0.05	0.69	[0.60, 0.79]
0.1	0.69	[0.58, 0.79]
0.2	0.64	[0.50, 0.67]

Confidence intervals computed via bootstrapping.

#### 5.5.2 Contribution of concept type

A crucial aspect of our approach is that concepts can be defined not only for objects but also for other aspects, such as task-specific concepts. In Task 1 (see Figure 3), for example, the concepts contain information about the objects (color and shape) as well as about the task (location). To show the importance of task-specific and object-specific annotations, we perform two additional trainings, each using only one of these subsets of the concepts.

As shown in Table 4, while both reduced concept sets improve over ACT, combining them gives the highest probability of improvement. This shows that the episode-level concept annotation in ConceptACT provides more benefit than auxiliary task information or object annotations alone.

**TABLE 4:** Probability of Improvement for object-only and task-only concept subsets compared to the full concept set (higher is better).

Method	Probability of improvement	95% CI
Over conceptACT with all concepts		
ConceptACT - object concepts	0.38	[0.29, 0.48]
ConceptACT - task rules	0.43	[0.34, 0.52]
Over prediction head with all concepts		
Prediction head - object concepts	0.32	[0.23, 0.39]
Prediction head - task rules	0.45	[0.36, 0.54]

#### 5.5.3 Other variants and baselines

In addition to the existing *Prediction Heads* variant, we evaluate two further concept integration methods. First, instead of structuring concepts into classes, we designed a “flat” concept transformer in which no class structure is built into the architecture. This changes the loss into a Binary Cross Entropy (BCE) loss. Second, to show the importance of the Concept Transformer architecture, we implemented a variant based on a Concept Bottleneck Model (Koh et al., 2020). Finally, to give the reader a second perspective, we also benchmark Diffusion Policy by Chi et al. (2025) as a comparison.

As shown in Table 5, the transformer architecture is crucial for success in learning. For the case of the class structure against a flat structure, the results are a bit more nuanced, providing a smaller benefit in improvements (but nevertheless significant).

**TABLE 5:** Probability of Improvement for the flat concept transformer, Concept Bottleneck Model, and Diffusion Policy baselines (higher is better).

Method	Probability of improvement (over conceptACT)	95% CI
Flat transformer	0.48	[0.38, 0.58]
Concept bottleneck model	0.06	[0.02, 0.10]
Diffusion policy	0.04	[0.01, 0.08]

### 5.6 Discussion

Our experimental results demonstrate that ConceptACT achieves significant improvements in both sample efficiency and learning efficiency compared to standard ACT. The method requires fewer demonstration episodes to reach target performance while accelerating convergence during training, addressing two primary cost factors in practical imitation learning deployments: human demonstration time and computational training resources.

While our evaluation focuses on two robotic manipulation tasks, the approach could extend to other imitation learning domain where meaningful high-level concepts can characterize episodes. Robotic manipulation provides a particularly suitable testbed because object properties, spatial relationships, and task constraints are naturally observable and semantically meaningful to human demonstrators. But the main idea of our approach, including episode-level concept annotation (which is cheap to collect) and using them as auxiliary supervision, can be extended to other domains. For example, in autonomous driving, concepts could represent weather, street conditions, or traffic conditions. Similar for (household) robots, concepts could be room types or also object categories.

In settings with structured collection protocols like ours, the practical annotation burden remains minimal. Demonstrators often naturally organize sessions around specific object types, environmental conditions, or task variants, collecting *“10 episodes with red cubes,” “5 episodes in cluttered environments,”* or *“15 episodes with deformable objects.”* Our episode-level concept annotations align with this existing structure: in our experiments, all concept labels were extracted automatically from the demonstration protocol without any manual labeling. Even in settings where automatic extraction is not possible, the manual annotation cost remains low. In a short experimental test with a simple command-line interface, annotating a single Task 1 episode took approximately 4 s compared to 8–14 s for performing the demonstration and about 15–30 s setup cost for each demonstration, adding roughly 3.5 min of total labeling time for a 50-episode dataset. But even when concepts are extracted automatic this does not remove the underlying dependency on humans completely. For each task, someone must still decide which concepts are relevant and how to group them, which ConceptACT does not discover automatically.

Our choice of episode-level rather than timestep-level concept annotation represents a deliberate trade-off between annotation cost and temporal specificity. While technically feasible to learn from concepts defined for individual timesteps or trajectory segments, such fine-grained annotation would dramatically increase the labeling burden and potentially introduce inconsistencies. Episode-level concepts capture the semantic invariants that remain constant throughout a demonstration while keeping annotation practical and scalable. For tasks requiring temporal concept variation within episodes, multiple shorter episodes would be more appropriate than fine-grained timestep labeling.

The better performance of Concept Transformer integration compared to simple prediction heads demonstrates that our improvements result not only from providing additional concept information, but specifically from the proposed architectural integration. If concept information alone drove the gains, the prediction head ablation would show similar performance to our full method, yet ConceptACT-Heads does not perform better than standard ACT, even in some cases worse. This stark difference validates that our architectural modification, though minimal since its only one layer, is crucial for effective concept utilization and that effective concept integration requires fundamental changes to how information flows through attention mechanisms rather than simply adding auxiliary prediction losses. On a similar note, the comparison with LAV-ACT shows comparable results: LAV-ACT, through its textual description, has access to semantic information and employs a significantly larger model architecture, yet ConceptACT achieves better performance with fewer parameters. This comparison also exposes a fundamental design trade-off. LAV-ACT’s language conditioning enables multi-task flexibility with the disadvantage that these textual inputs (which include the semantic information) are required at every deployment episode, a cost that scales linearly with the number of deployments. ConceptACT does not incur this scaling cost: concept annotations are a one-time fixed cost during demonstration collection, after which the policy deploys on standard sensorimotor inputs alone. That ConceptACT achieves superior performance despite this strictly lighter deployment requirement indicates that structured concept integration through attention mechanisms provides more effective inductive biases than conditioning on unstructured language, even when that language contains comparable semantic content.

## 6 Conclusion

We introduced ConceptACT, an extension of *Action Chunking with Transformers (ACT)* that incorporates episode-level concepts into imitation learning. We achieve this by allowing the human demonstrator to provide concepts during data collection. These concepts are used as an auxiliary supervision signal only during training (deployment does not require additional concept information). With this, we have significant improvements in both sample efficiency and learning speed compared to standard ACT.

Our key finding is that how concepts are integrated matters more than simply having access to them. Our approach, which changes the classical concept transformer to include class information, substantially outperforms both naive concept prediction and language-conditioned models despite using fewer parameters. This shows that not only is the concept information important, but also its structural integration is important.

These results have direct practical implications: ConceptACT can reduce demonstration requirements by annotating episodes with observable concepts during data collection. This differs from approaches like LAV-ACT (or other language-conditioned approaches), which require the specification of concepts through language at deployment time, thereby increasing deployment costs.

While our evaluation focused on manipulation tasks with discrete concepts, the principle of including human concept understanding during training could extend to other domains where episodes can be (partially) categorized by their concepts. In addition to the evaluation across different settings, Possible future work could include investigating methods for automatic concept discovery. In addition, it could be examined how the concept-guided learning changes the learned representations and if these changes make the policy more interpretable.

While there is still a gap between how humans and robots learn tasks, ConceptACT demonstrates that this gap can be reduced. This is achieved by allowing humans to teach not just through actions but also through the conceptual understanding they naturally possess, aligning robot learning more closely with human learning while remaining practically deployable.

## Data Availability

The raw data supporting the conclusions of this article will be made available by the authors, without undue reservation.

## Footnotes

1 We keep using a VAE prefix for the Encoder and Decoder here, to minimize naming confusion for later parts of the architecture, where there is also a transformer-based encoder and decoder.

2 Please note that sequence here means in terms of patches/token extracted from the input states & images. Not a sequence in the demonstration dataset.

3 We apply label smoothing with ϵ=0.1 to prevent overconfident predictions on the one-hot targets.
